# Invalid-Resource-Aware Spectrum Assignment for Advanced-Reservation Traffic in Elastic Optical Network

**DOI:** 10.3390/s20154190

**Published:** 2020-07-28

**Authors:** Shufang He, Yang Qiu, Jing Xu

**Affiliations:** 1 Key Laboratory of State Ethnic Affairs Commission for Electronic and Information Engineering, College of Electrical & Information Engineering, Southwest Minzu University, Chengdu 610041, China; shufang_hh@163.com; 2 Optical Communications Laboratory, Ocean College, Zhejiang University, Zheda Road 1, Zhoushan 316021, China; jxu-optics@zju.edu.cn

**Keywords:** elastic optical network, invalid spectrum rate, advanced reservation, defragmentation, blocking probability, spectrum alignment rate

## Abstract

Elastic optical networks (EONs) can make service accommodation more flexible and precise by employing efficient routing and spectrum allocation (RSA) algorithms. In order to improve the efficiency of RSA algorithms, the advanced-reservation technique was introduced into designing RSA algorithms. However, few of these advanced-reservation-based RSA algorithms were focused on the unavailable spectrum resources in EONs. In this paper, we propose an Advanced-Reservation-based Invalid-Spectrum-Aware (AR-ISA) resource allocation algorithm to improve the networking performance and the resource alignment of EONs. By employing a new index, Invalid Spectrum Rate (ISR), to record the proportion of unavailable spectrum resources in EONs, the proposed AR-ISA algorithm set a network load threshold to trigger the postponement of an arriving service. Compared with the traditional slack-based AR mechanism, the proposed algorithm has more concerns about the current spectrum usage of the path designated by the service than the conflicts between AR services and other existing services. To further increase the networking performance, the proposed algorithm adopts defragmentation to increase the number of available spectrum resources when postponing a service. Theoretical analysis and simulation results show that the proposed AR-ISA algorithm has obvious effectiveness in reducing the service blocking rate and increasing the spectrum alignment rate.

## 1. Introduction

Recently, with the emergence and development of a variety of novel network services, such as high definition video delivering and cloud computing, network services have become varied and require more bandwidth. All these increase people’s demands for heterogeneous kinds of data transmission, especially for high-speed ones, and the network traffic grows rapidly as a result [1]. Optical network technologies have been considered as a promising solution to high-rate transmission. However, the traditional wavelength division multiplexing (WDM) optical network can hardly adapt to diverse service requests due to its intrinsic limitation in spectrum allocation. Since the WDM optical network allocates constant wavelength to different services with diverse bandwidth requirements, it lacks flexibility in resource allocation and has a limited spectrum utilization [2]. Therefore, the elastic optical network (EON) technique has been proposed to increase the flexibility and efficiency of resource allocation [3]. By employing orthogonal frequency division multiplexing technology [4], EON breaks the rigid allocation limitations of the traditional WDM network and can allocate adaptive spectrum resources to different services according to their individual bandwidth requirements. This can greatly improve the flexibility and efficiency of an optical network.

The routing and spectrum assignment (RSA) algorithm is the key technology to realize the flexibility and efficiency of EONs [5,6,7,8,9]. Therefore, many sophisticated RSA algorithms have been proposed for EONs [10,11,12,13,14,15,16,17,18,19,20]. In [10,11], researchers introduced classic routing strategies and typical resource allocation algorithms, such as the shortest-path-routing strategy and first-fitting or random fitting mechanisms into the RSA design. In [12,13], distance-adaptive modulation techniques were introduced into RSA algorithms to allow the flexible selection of a modulation format for a service request according to its transmission distance. In [14], minimum and maximum entropy-based RSA algorithms were introduced into EONs, which brought about improvement in supporting statically growing services and reduced spectrum fragments. In [15], W.B. Jia et al. applied the prediction of reverse neural network to improve the efficiency of the RSA and reduced the blocking rate of EONs. In [16], the awareness of Quality-of-Transmission was considered in the RSA algorithm to enhance the performance of EONs.

However, these typical RSA algorithms ignored some small, isolated, and unoccupied spectrum bands (a.k.a. spectrum fragments) which remained after the allocation of spectrum resources in EONs [17]. Since the spectrum fragments could hardly be utilized by the subsequent services due to the constraints in resource allocation and thus greatly affected the utilization efficiency of EONs, many spectrum defragmentation algorithms were proposed [18,19,20,21,22,23,24,25,26,27,28,29,30]. In [18], a hitless spectrum retuning technique was introduced into the defragmentation algorithm. In [19], a multipath technique was employed to improve the efficiency of the defragmentation algorithms. A push-pull defragmentation algorithm [20] and priority-based defragmentation algorithm [21] were proposed to increase the efficiency of the spectrum defragmentation in EONs. In [22], the researchers solved the problem of serial spectrum defragmentation by employing a dependency graph, which reduced the network spectrum fragments through the original routing and spectrum optimization scheme, and then spectrum defragmentation was performed by the dependency graph. In [23], the authors sorted the free spectrum blocks in the network and selected a spectrum block with a smaller size to provision the existing service so that the spectrum fragments in the network could be reduced. In [24,25], schemes based on an auxiliary graph were proposed to solve the problem of spectrum defragmentation, in which the problem was transformed into the problem of finding the largest independent set in the constructed auxiliary graph. Besides this, fragmentation-aware RSA algorithms [26,27] and group-based RSA algorithms [28,29,30,31] were proposed to reduce the spectrum fragments generated in EONs so as to increase their efficiency in resource utilization. Additionally, in [32,33] the survivability issue was considered in designing RSA algorithms. 

Although the above RSA algorithms succeeded in increasing the allocation flexibility and utilization efficiency of EONs, most of them neglected the timing characteristics of the service requests in EONs. Thus, the advanced-reservation technique [34] was introduced into RSA algorithms to improve their efficiency in time-scheduling, so that the arriving services can be accommodated at the optimal time and the spectrum resources can be further optimized. Thus, many research works have been carried out on the advanced-reservation-based RSA algorithms [35,36,37,38,39,40,41,42]. In [35], the researchers proposed the concept of spectrum resource usage (SRU), and considered the time domain and frequency domain simultaneously to minimize SRU for the statically scheduled AR requests. Similar to [35], the researchers in [36] introduced a parameter to measure each available frequency-time block of AR requests. Besides this, dynamic scheduling methods were adopted in [37,38,39]. In [37], the notion of the degree of spectrum-time fragmentation (DSTF) was adopted in dynamic scheduling. In [38], the researchers solved the conflict between the AR and other traditional requests during scheduling. In [39], the request scheduling in the time domain was investigated in addition to the routing, modulation, and spectrum assignment (RMSA). In [40], the researchers proposed an advanced-reservation technique based on flexible time-windows. They explored the effects of different request time-windows and service times without the consideration of time-slots. In [41], a slack-based new mechanism was adopted to allocate resources flexibly in EONs. In such a mechanism, the client specified the slack time and the reservation of resources was carried out within the start time and the slack time. With this approach, flexible service scheduling can be realized. Besides this, in order to describe the spectrum occupation in the time axis, the authors in [42] introduced a concept of Time-Spectrum Consecutiveness (TSC). By calculating the value of TSC for all the arrival times and deadline times and selecting the maximum TSC, the provisioning time and the routing path were optimized for spectrum allocation in EONs. However, the above-mentioned AR algorithms ignored the investigation of the unavailable spectrum resources in EONs, which may have a negative effect on the decision of provisioning time for a service request and thus affect the optimization of the spectrum resources as well as the networking performance (e.g., service blocking probability).

In this paper, we propose an AR-ISA algorithm to improve the networking performance (e.g., service blocking probability) and resource alignment in EONs. By employing a new index, invalid spectrum rate (ISR), to record the proportion of unavailable spectrum resources (including both the occupied and the fragmented ones) in a path, the proposed AR-ISA algorithm set a network load threshold to trigger the postponement of an arriving service. Compared to the traditional slack-based AR mechanism, the proposed algorithm has more concerns about the current spectrum usage of the path selected by the service than the conflicts between the AR services and other existing services. To further increase the networking performance, the proposed algorithm adopts spectrum defragmentation to increase the number of available spectrum resources when postponing a service. The simulation results show that the proposed AR-ISA algorithm helps reduce the service blocking rate and increase the spectrum alignment rate.

The rest of this paper is organized as follows. In Section 2, a network model is introduced for the proposed AR-ISA algorithm. In Section 3, the details of the proposed AR-ISA algorithm are analyzed. In Section 4, the performance of the proposed AR-ISA algorithm is investigated. Finally, this paper is summarized in Section 5.

## 2. The Network Modeling for the Proposed AR-ISA Algorithm

In this section, we introduce the network model for the proposed AR-ISA algorithm. Firstly, we define the physical topology of an elastic optical network as *G*(*V*,*E*,*S*), in which *V* represent the set of network nodes, *E* represents the set of network links, and *S* represents the set of frequency slots (FSs) on each link. For simplicity, we represent the values of *|V|*, *|E|*, *|S|* as *N*, *L*, *F*, respectively.

In order to investigate the status of all the FSs in the network, we define a two-dimensional array as follows:

(1) $$U=\left[\begin{array}{cc} \begin{array}{ccc} u_{1}^{1} & u_{2}^{1} & \cdots \\ u_{1}^{2} & u_{2}^{2} & \cdots \\ \vdots & \vdots & \ddots \end{array} & \begin{array}{c} u_{i}^{1}\\ u_{i}^{2}\\ \vdots \end{array}\\ \begin{array}{ccc} u_{1}^{l} & u_{2}^{l} & \cdots \end{array} & u_{i}^{l} \end{array}\right].$$

In (1), $u_{i}^{l}$ indicates whether the *i*th FS on the *l*th link is occupied, where I ∈F and l∈L. When $u_{i}^{l}=1$, it indicates that the corresponding FS has been occupied and cannot be allocated to other services until it is released. In contrast, $u_{i}^{l}=0$ indicates that the FS is unoccupied. Thus, we can calculate the quantity of the occupied FSs along a path p via the following equation:

(2) $$U_{op}=\sum u_{i}^{l},$$

where *p*(*s*,*d*) indicates the set of the links along the path *p* for a service, with s and d representing the source and the destination nodes of the service, respectively.

Given the spectrum continuity and contiguity constraints in the resource allocation, EONs can calculate the required amount of FSs for an arriving service request. More specifically, by setting one FS as 12.5 GHz and employing the distance-adaptive modulation technique, the amount of FSs required by a service *M* (including one guard band) with data rate *C* can be calculated as follows:

(3) $$M=\left\lceil \frac{C}{m\times 12.5}\right\rceil +1,$$

where *m* represents the modulation level of the service. Noticeably, the values of *m* can be selected from 1~4 based on the relations between the modulation formats and their corresponding transmission ranges, as in the following Table 1 [43].

Note that, due to the spectrum continuity and contiguity constraints in resource allocation, some isolated, unoccupied spectrum bands remain. These remained spectrum bands cannot provide enough available FSs for the subsequent services and thus are known as spectrum fragments. In this paper, we define the spectrum fragments as the unoccupied spectrum bands that contain contiguous FSs less than the minimum value of M, M_min_. The spectrum fragments along a path *p*, *U_fp_*, can be defined as the sum of all spectrum bands containing continuous vacant FSs less than M_min_ along the path.

Given the unavailability of both the occupied and fragmented FSs in spectrum allocation, we use the new index, ISR, to calculate the proportion of both the occupied and the fragmented FSs along path *p* to all spectrum resources of this path as follows:

(4) $$ISR_{p}=\frac{U_{op}+U_{fp}}{F\cdot L(s,d)},$$

where *L*(*s*,*d*) is the number of all links included in path *p*.

In this paper, we propose the AR-ISA algorithm to improve the networking performance and the resource alignment in EONs according to the above-introduced index, ISR. When the calculated ISR exceeds a pre-set maximum value, network postponement will be triggered by the proposed algorithm. In order to further reduce the amount of unavailable spectrum resources, when a service is about to be blocked spectrum defragmentation is triggered to reduce the fragmented FSs. Since the proposed algorithm can effectively reduce the amount of invalid FSs, more available spectrum resources can be remained for the subsequent service requests. In this way, the service blocking performance can be improved.

## 3. Details of the Proposed Advanced-Reservation-Based Invalid-Spectrum-Aware Resource Allocation Algorithm

**Figure 1:**
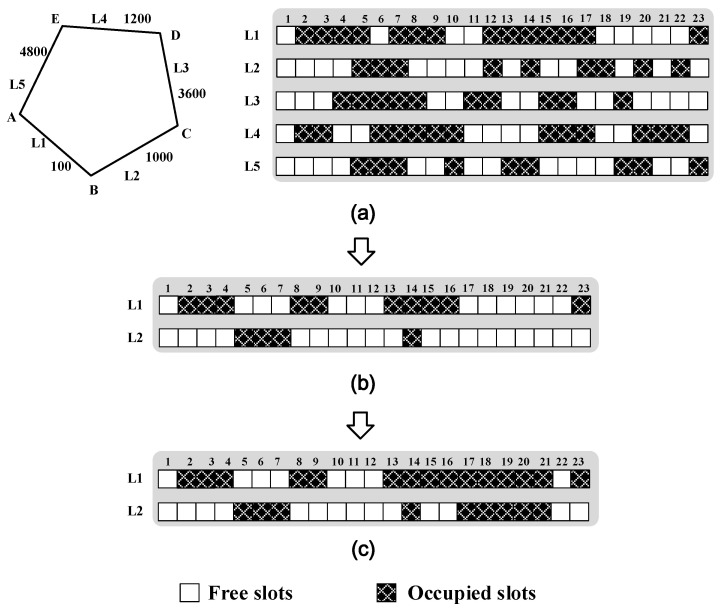
An illustrative example for the proposed Advanced-Reservation-based Invalid-Spectrum-Aware (AR-ISA) algorithm. (**a**) The network topology employed and the usage of all frequency slots (FSs) at a certain time, (**b**) after FSs are released on links L1 and L2, (**c**) available FSs on links L1 and L2 found for the service.

In this section, we illustrate the details of the proposed advanced-reservation-based invalid-spectrum-aware resource allocation algorithm. For a more intuitive understanding of the formulas listed above, a simple example is adopted to illustrate the proposed AR-ISA algorithm as in Figure 1. As shown in Figure 1a, the adopted network consists of five nodes and five bidirectional links, with the distance of each link marked. The usage of all the FSs at a certain time is also shown in Figure 1a, assuming that there are totally 23 FSs on each link. In the example, we assume that a new service R1(A, C, 200) arrives, where A and C are its source and destination nodes, respectively, and 200 (in the unit of bit/s) is its required data rate. By selecting the links, L1 and L2, as its routing path, R1 needs to transmit 1100 km from A to C. Therefore, its highest modulation level can be 4 and it requires $M=\frac{200}{4\times 12.5}+1=5$ FSs accordingly. When its modulation level is selected as 1, 2, or 3, its required FSs are 7, 9, or 17, respectively. Thus, we regard the unoccupied FS bands that contain less than 5 FSs as fragmented FSs. The amount of the fragmented FSs can be calculated as $U_{fp}=4+14=18$ in links L1 and L2. Besides this, the amount of the occupied FSs can be calculated as $U_{op}=15+9=24$. Thus, the value of ISR_p_ is $\frac{U_{op}+U_{fp}}{F\cdot L(s,d)}=\frac{24+18}{23\times 2}=0.913$. Not enough available FSs on links L1 and L2 can be found for R1 at the moment, as shown in Figure 1a. By assuming to trigger the postponement of a service request with anISR_p_ larger than 0.7 in this illustrative example, the proposed algorithm may postpone R1 and some occupied FSs may be released, as in Figure 1b. Then, the available FSs on links L1 and L2 can be found for R1, as shown in Figure 1c. Noticeably, spectrum defragmentation can be triggered if there are still unavailable FSs found after postponing services.

The details of the proposed AR-ISA algorithm can he found in the following Algorithm 1. The proposed algorithm calculates K shortest paths for each node pair in the network and sets a maximum invalid spectrum ratio (MISR) before any service arrives. For simplicity, we use R(s, d, B, t_h_) to represent a service request, in which *s* and *d* represent the source and destination nodes of R, respectively; B is the data rate of R; and t_h_ is the serving duration of R. When a new service R(s, d, B, t_h_) arrives, K pre-computed paths are selected as candidate paths for R, and the modulation level as well as the amount of the needed FSs on each path is calculated according to Equation (3). For each candidate path, the proposed algorithm calculates the ISR of the path to trigger the postponement of R if the calculated ISR is larger than the preset MISR. Then, the proposed algorithm tries to seek available FSs on the path. If any available FSs are found on any candidate path, we accommodate R according to the first-found available FSs and their corresponding path. Otherwise, spectrum defragmentation is triggered for R before the spectrum resources on the K candidate paths are investigated again. If no available FSs are found for R, it will be blocked. Otherwise, it will be accommodated by the first-found available FSs. Note that the postpone time in the proposed algorithm is related to the time interval between two successive services.
**Algorithm 1.** Details of the proposed AR-ISA algorithm.**AR-ISA Algorithm**1: Compute K shortest-paths for each node pair in the network
2: Set a MISR for the algorithm
3: **while** network is running **do**
4: When a service R arrives
5:  Select K pre-computed paths for R as its candidate paths
6:  Calculate the modulate level and the amount of the needed FSs on each candidate path for R
7:  **for** one path p in the K candidate paths **do**
8:   Calculate the ISR along p
9:   **If** the calculated ISR is larger than pre-set MISR **then**
10:    Postpone R according to the time interval between two successive services
11: **end if**
12:   Find available FSs on P for R
13: **if** available FSs are found on p **then**
14: Allocate the found FSs to R
15:   **end if**
16: **break**
17: end for
18:  **If** spectrum allocation for R is unsuccessful **then**
19:   Do spectrum fragmentation for the existing services
20: **for** one path pin the K candidate paths **do**
21: Find available FSs on P for R
22: **if** available FSs are found on p **then**
23: Allocate the found FSs to R
24: end if
25:  **break**
26: **end for**
27: **end if**
28:  **If** spectrum allocation for R is unsuccessful **then**
29:   Block R
30:  **end if**
31:  Update the network
32: **end while**

The time complexity of the AR-ISA algorithm is mainly determined by its adopted path routing algorithm and spectrum allocation strategy. As for the path routing algorithm, the proposed algorithm employs the Dijkstra algorithm and link deleting strategy to find K shortest paths for each node pair. Thus, its complexity can be calculated as *O(K×|E|×|V|^2^)*, with *|E|* and *|V|* representing the amounts of links and nodes in the network. As for the spectrum allocation procedure, its time complexity is mainly determined by the process of finding available FSs for an arrival service and the possible spectrum defragmentation for the existing services at the worst case, and thus can be calculated as *O(2×K×|E|^2^×|S|^2^ + N×K×|E|^2^×|S|^2^)*, where *N* represents the amount of existing services and *|S|*represents the amount of FS on a link. In general, the time complexity of the proposed AR-ISA algorithm is a polynomial. The acronyms used in this paper are summarized in the following Table 2.

## 4. Performance Evaluation

**Figure 2:**
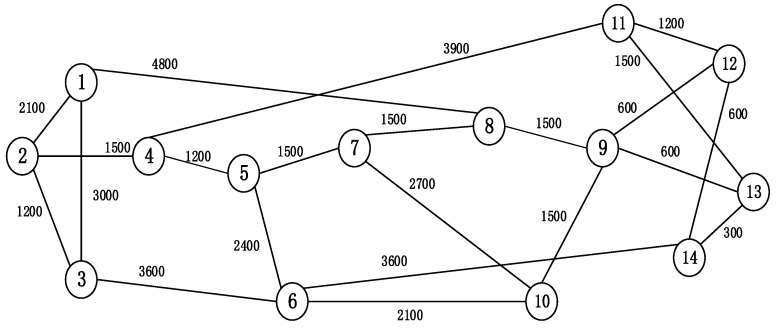
National science foundation network (NSFNET) topology.

In this section, we perform simulations in DEV-C++to evaluate the effectiveness of the proposed AR-ISR algorithm in the typical national science foundation network (NSFNET), as in Figure 2. In the network, we assume that each link consists of two unidirectional fibers, with each one containing 320 FSs. We also assume that four kinds of services with different required data rates—namely, 50 Gb/s, 100 Gb/s, 150 Gb/s, and 200 Gb/s—are accommodated by the network. Their proportion is set as 1:1:1:1. In order to emulate the dynamic traffic generation, a Poisson traffic model is adopted in the simulation. In the model, a parameter λ is used to emulate the arrival rate of the services, and a negative exponential distribution with parameter μ is used to emulate the duration times of the services. We use the formula *λ/μ* to indicate the traffic load in the network, as in [44]. In the generation of each service, its source and destination, as well as its data rate, are randomly determined from their sample spaces. Noticeably, in this simulation Formula (3) can be used to calculate that the number of FSs required by various services is 2,3,4,5,7,9,13, and 17. Table 3 summarizes all the parameter settings in the simulations.

**Figure 3:**
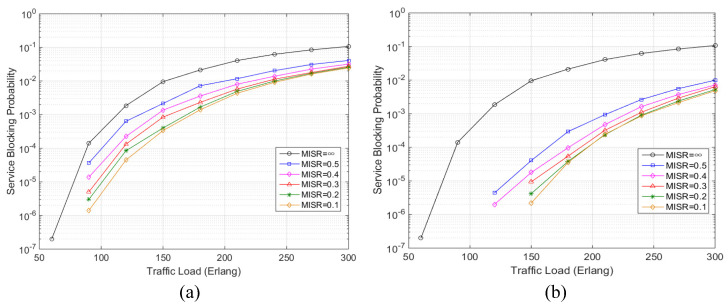
Simulation results of the service blocking probability. (**a**) Simulation results on the service blocking probability vs. traffic load with different values of maximum invalid spectrum ratio (MISR) when no spectrum defragmentation is adopted, (**b**) simulation results on the service blocking probability vs. traffic load with different values of MISR when spectrum defragmentation is adopted.

**Figure 4:**
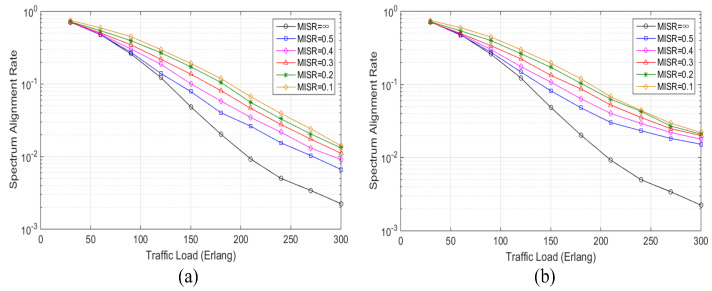
Simulation results of the spectrum alignment rate. (**a**) Simulation results of the spectrum alignment rate vs. the traffic load with different values of MISR when no spectrum defragmentation is adopted, (**b**) simulation results of the spectrum alignment rate vs. the traffic load with different values of MISR when spectrum defragmentation is adopted.

In order to verify the effectiveness of the proposed AR-ISR algorithm in NSFNET, the proposed algorithm and an AR algorithm based on ISR without defragmentation, are investigated in the simulation. In the simulation, we set the delayed time as 0.5 times the arrival interval between two services, which is randomly generated according to the above-mentioned Poisson model. Figure 3 to Figure 4 illustrate the simulation results of the proposed algorithm on the service blocking probability and spectrum alignment rate when the MISR changes from 0.1 to 0.5. In the simulation, the service blocking probability is defined as the ratio of blocked services to all the generated services, while the spectrum alignment rate is defined as the proportion of vacant FSs with the same index on all links. Specially, the curve MISR = ∞ represents the traditional distance-adaptive routing and spectrum allocation algorithm, as in [10].

Figure 3a shows the simulation results of the service blocking probability with different values of MISR when no spectrum defragmentation is adopted in the proposed AR-ISR algorithm. By using MISR = ∞ to represent the classic distance-adaptive RSA algorithm [12], the proposed AR-ISR algorithm (with no spectrum defragmentation) gains an obvious advantage over the classic distance-adaptive algorithm in reducing the service blocking probability, as shown in Figure 3a, although such an advantage may come to saturation with the MISR decreasing from 0.5 to 0.1. For instance, the proposed AR-ISR algorithm with no spectrum defragmentation can reduce the service blocking probability by 77.55% compared to the distance-adaptive algorithm, with MISR being set as 0.5 when the traffic load is 150 Erlang. Such a reduction in the service blocking probability reaches 96.49%, with the MISR being set as 0.1 when the traffic load is 150 Erlang. As for the high traffic load (e.g., 300 Erlang), the proposed AR-ISR algorithm with no spectrum defragmentation can still reduce the service blocking probability by 61.76% compared to the distance-adaptive algorithm with the MISR being set as 0.5. Such a reduction in the service blocking probability reaches 77.03% with the MISR being set as 0.1. All the above-mentioned simulation results indicate that the proposed AR-ISR algorithm can effectively reduce the amount of blocked services by postponing the accommodation of a service according to invalid spectrum rate in the network, even without any spectrum defragmentation. It can be understood by the fact that by employing the invalid-resource-aware mechanism, the proposed AR-ISR algorithm paves a way to optimize the service scheduling and thus increases the networking performance due to the increased number of available FSs.

Figure 3b shows the simulation results of the service blocking probability with different values of MISR when spectrum defragmentation is adopted in the proposed AR-ISR algorithm. Similar to Figure 3a, the proposed AR-ISR algorithm gains an obvious advantage over the classic distance-adaptive algorithm in reducing the service blocking probability, although such an advantage may come to saturation with the MISR decreasing from 0.5 to 0.1. For instance, the proposed AR-ISR algorithm (with spectrum defragmentation) can reduce the service blocking probability by 99.55% compared to the distance-adaptive algorithm, with the MISR being set as 0.5 when the traffic load is 150 Erlang. Such a reduction in the service blocking probability reaches 99.97%, with the MISR being set as 0.1 when the traffic load is 150 Erlang. When the traffic load reaches as high as 300 Erlang, the proposed AR-ISR algorithm (with spectrum defragmentation) can still reduce the service blocking probability by 90.50% compared to the distance-adaptive algorithm, with the MISR being set as 0.5. Such a reduction in the service blocking probability reaches 95.47% with the MISR being set as 0.1. The above simulation results verify that by employing the spectrum defragmentation mechanism, the proposed AR-ISR algorithm can further reduce the amount of blocked services by reducing the unavailable spectrum resources and improve the service blocking performance of the network. All the simulation results in Figure 3a,b verify that the proposed AR-ISR algorithm can effectively reduce the service blocking probability by adopting invalid resource awareness in service scheduling and spectrum defragmentation in service allocation.

Figure 4a depicts the simulation results of the spectrum alignment rate performance with different values of MISR when no spectrum defragmentation is adopted in the proposed AR-ISR algorithm. By using MISR = ∞ to represent the classic distance-adaptive RSA algorithm [12], the proposed AR-ISR algorithm (with no spectrum defragmentation) gains an obvious advantage over the classic distance-adaptive algorithm in increasing the spectrum alignment rate, especially with a high traffic load, as shown in Figure 4a. For instance, when the traffic is as low as 90 Erlang, the proposed AR-ISR algorithm (with no spectrum defragmentation) can enhance the spectrum alignment rate by 5.34% compared to the distance-adaptive algorithm, with the MISR being set as 0.5. Such an improvement in the spectrum alignment rate reaches 72.7% with the MISR being set as 0.1. When the traffic load reaches as high as 300 Erlang, the proposed AR-ISR algorithm (with no spectrum defragmentation) can increase the spectrum alignment rate by 196.77% compared to the distance-adaptive algorithm, with the MISR being set as 0.5. Such an improvement in spectrum alignment rate reaches 528.67% with the MISR being set as 0.1. All the simulation results indicate that the proposed AR-ISR algorithm can effectively improve the spectrum alignment rate by reducing the invalid spectrum resources even without spectrum defragmentation, especially with a high traffic load. The higher improvement in spectrum alignment rate with a higher traffic load can be understood by the fact that more occupied and fragmented FSs are generated with higher traffic loads, which makes the services’ postponement more likely.

Figure 4b depicts the simulation results of the spectrum alignment rate performance with different values of MISR when spectrum defragmentation is adopted in the proposed AR-ISR algorithm. Similar to Figure 4a, the proposed AR-ISA algorithm (with spectrum defragmentation) gains an obvious advantage over the classic distance-adaptive algorithm in increasing the spectrum alignment rate, especially with a high traffic load. For instance, when the traffic is as low as 90 Erlang, the proposed AR-ISA algorithm (with spectrum defragmentation) can enhance the spectrum alignment rate by 6.37% compared to the distance-adaptive algorithm, with the MISR being set as 0.5. Such an improvement in the spectrum alignment rate reaches 71.12% with the MISR being set as 0.1. When the traffic load reaches as high as 300 Erlang, the proposed AR-ISA algorithm (with spectrum defragmentation) can increase the spectrum alignment rate by 580.32% compared to the distance-adaptive algorithm, with the MISR being set as 0.5. Such an improvement in the spectrum alignment rate reaches 881.6% with the MISR being set as 0.1. All the simulation results imply that the proposed AR-ISA algorithm can improve the spectrum alignment rate more effectively by employing the spectrum defragmentation mechanism to reduce the fragmented FSs, especially with a high traffic load. All the simulation results in Figure 4a,b verify that the proposed AR-ISA algorithm can effectively improve the spectrum alignment rate by adopting invalid resource awareness in service scheduling and spectrum defragmentation in service allocation. Noticeably, fiber nonlinearities may distort the performance of the proposed AR-ISA algorithm in EONs, especially for high-rate long-range transmission [45]. Some nonlinear compensation techniques, such as full-field nonlinear compensation with probabilistically shaped constellations [46], single-channel digital back-propagation [47], optical phase conjugation [48], nonlinearity-tailored detection [49], and nonlinear Fourier transform [50], can be adopted in the EONs to guarantee the effectiveness of the proposed AR-ISA algorithm.

## 5. Conclusions

In this paper, we propose an AR-ISA algorithm to improve the networking performance and the resource alignment of EONs. By employing a new index, ISR, to record the proportion of unavailable spectrum resources (including both the occupied and the fragmented ones) along a path, the proposed AR-ISA algorithm set a network load threshold to trigger the postponement of an arriving service. Compared to the traditional AR algorithm, the proposed algorithm has more concerns about the current spectrum usage of the path the service selected. To further optimize the unavailable resources, the proposed algorithm adopts defragmentation to increase the number of available spectrum resources when postponing a service. The simulation results verify that by employing ISR to optimize the postponement of the service accommodation and the defragmentation technique to increase the available FSs, the proposed AR-ISA algorithm improves the service blocking performance and the spectrum alignment rate.

## Figures and Tables

**Table 1:** Modulation formats vs. their corresponding transmission range.

Transmission Range (km)	Modulation Level(bit/Hz)	Modulation Format
9600	1	BPSK
4800	2	QPSK
2400	3	8-QAM
1200	4	16-QAM

**Table 2:** A summary of acronyms.

Full Name	Abbreviation
EONs	Elastic optical networks
RSA	Routing and spectrum allocation
AR-ISA	Advanced-reservation-based invalid-spectrum-aware
ISR	Invalid spectrum rate
WDM	Wavelength division multiplexing
SRU	Spectrum resource usage
DSTF	Degree of spectrum-time fragmentation
RMSA	Routing, modulation and spectrum assignment
TSC	Time-spectrum consecutiveness
FSs	Frequency slots
MISR	Maximum invalid spectrum ratio
NSFNET	National science foundation network

**Table 3:** Parameter settings in simulations.

Parameters	Settings
Network topology	NSF-Net
K, number of candidate paths	3
F, number of frequency slots	320
Bandwidth of one frequency slot	12.5 GHz
Number of guard band	1
Optional modulation formats	BPSK, QPSK, 8-QAM, 16-QAM
Bits per symbol of different modulation formats	1, 2, 3, 4
Reachable distance of different modulation formats (km)	9600, 4800, 2400, 1200
Type of services (Gb/s)	50, 100, 150, 200
Proportion of different types of services	1:1:1:1
Arrival rate of services	Poisson distribution model
Duration time of each service	Negative exponential distribution
Number of services per simulation	5 × 10^6^
